# Temperature-Regulated IncX3 Plasmid Characteristics and the Role of Plasmid-Encoded H-NS in Thermoregulation

**DOI:** 10.3389/fmicb.2021.765492

**Published:** 2022-01-06

**Authors:** Liu Baomo, Shui Lili, Robert A. Moran, Willem van Schaik, Zhuo Chao

**Affiliations:** ^1^State Key Laboratory of Respiratory Disease, The First Affiliated Hospital of Guangzhou Medical University, Guangzhou, China; ^2^Department of Respiratory and Critical Care Medicine, The First Affiliated Hospital of Sun Yat-sen University, Guangzhou, China; ^3^Department of Pulmonary and Critical Care Medicine, The First Affiliated Hospital of Chongqing Medical University, Chongqing, China; ^4^Institute of Microbiology and Infection, University of Birmingham, Birmingham, United Kingdom

**Keywords:** IncX3, carbapenem resistance Enterobacteriaceae, temperature, plasmid-encoded hns, fitness

## Abstract

Carbapenem-resistant Enterobacteriaceae (CRE) are a critical public health problem worldwide. Globally, IncX3-type plasmids have emerged as the predominant vehicles carrying the metallo-β-lactamase gene *bla*_NDM_. Although *bla*_NDM_-bearing IncX3 plasmids have been found in various hosts from diverse environments, whether their transfer and persistence properties vary under different conditions and what factors influence any variation is unknown. By observing the effects of different temperatures on IncX3 plasmid conjugation rates, stability, and effects on host fitness in *Escherichia coli*, we demonstrate that temperature is an important determinant of plasmid phenotypes. The IncX3 plasmid pGZIncX3 transferred at highest frequencies, was most stable and imposed lower fitness costs at 37°C. Temperature-regulated variation in pGZIncX3 properties involved a thermoregulated plasmid-encoded H-NS-like protein, which was produced at higher levels at 30°C and 42°C and inhibited the expression of type IV secretion system genes involved in conjugation. These findings suggest that *bla*_NDM_-bearing IncX3 plasmids are adapted to carriage by enterobacteria that colonize mammalian hosts and could explain the rapid dissemination of these plasmids among human-associated species, particularly in hospital settings.

## Introduction

Carbapenem-resistant Enterobacteriaceae (CRE) are a critical public health problem globally, as they can exhibit resistance to almost all available antibiotics and are responsible for extensive morbidity and mortality (Logan and Weinstein, 2017). Bacteremia due to CRE can result in mortality rates higher than 40% in China (Zhang et al., 2018). New Delhi metallo-β-lactamase (NDM)-type carbapenemase is the major mechanism mediating carbapenem resistance in approximately 40% of CRE isolates (Bush and Bradford, 2020). Although the *bla*_NDM_ gene can be found inserted in the chromosome, the substantial majority of carriage is linked to plasmids, which contribute significantly to the rapid dissemination of carbapenem resistance (Khan et al., 2017).

IncX3 plasmids are emerging as the predominant plasmid type carrying *bla*_NDM_ worldwide, with approximately one third of *bla*_NDM_-carrying plasmids deposited in GenBank containing IncX3-type replicons (Ma et al., 2020). Additionally, IncX3 plasmids appear to function as a platform for the evolution of NDM enzymes, as diverse *bla*_NDM_ gene variants have been found in plasmids of the same replicon type (Ma et al., 2020). Strains containing *bla*_NDM_-bearing IncX3 plasmids have been isolated from clinical settings (Zhou et al., 2020), companion animals (Nigg et al., 2019), agricultural settings (Zhang et al., 2019), and the environment (Zhai et al., 2020), which suggests they can persist in their hosts under various conditions, including different temperatures. The mechanisms that facilitate IncX3 plasmid persistence and dissemination at different temperatures are unknown.

In *Escherichia coli* and other Gram-negative bacteria, the histone-like nucleoid structuring (H-NS) protein has been shown to take part in the regulation of chromosomal transcriptional networks (Dorman and Ni Bhriain, 2020). Many genes encoding different H-NS family proteins have been found in microbial chromosomes as well as in plasmids (Shintani et al., 2015). Our previous work revealed that a H-NS protein homolog encoded by an IncX3-plasmid inhibits plasmid transfer and partitioning (Liu et al., 2020). Plasmid-encoded H-NS-like proteins are also known to play an important role in the regulation of transfer genes in IncH plasmids. The well-studied IncHI1 plasmid R27 exhibits thermosensitive variation in conjugative transfer frequencies modulated by host- and plasmid-encoded H-NS-like proteins (Forns et al., 2005).

In this study, we examined the IncX3 plasmid-encoded H-NS-like protein and characterized its effect on plasmid phenotypes at various temperatures. We show that this protein is essential in linking plasmid phenotypes with temperatures.

## Materials and Methods

### Bacterial Strains, Plasmids, and Culture Conditions

Bacterial strains and plasmids presented in this study are detailed in Table 1.

**Table 1:** All test strains in this work.

Strains	Description	Reference
J53	*E. coli* F^−^ *met pro* Azi^r^	Matsumura et al., 2018
J330	J53 with pGZIncX3 transferred naturally	Liu et al., 2020
J330(*phns*)	J53 with pACYC184*phns* transferred by electroporation	This work
J330(pACYC184)	J53 with pACYC184 transferred by electroporation	This work
Pkhns	J53 with pGZIncX3Δ*phns* transferred by electroporation	Liu et al., 2020
Pkhns(*phns*)	J330 with pACYC184 *phns* transferred by electroporation	This work
Pkhns(pACYC184)	J330 with pACYC184 transferred by electroporation	This work
Ec600	*E.coli* recipient strains for conjugation experiments	Laboratory stock
**Plasmids**		
pGZIncX3	blaNDM^+^, IncX3, sharing 99% similarity with pNDM-HN380	Liu et al., 2020
pGZIncX3Δ*phns*	pGZIncX3 knocking down plasmid-encoded phns gene	Liu et al., 2020
pACYC184	Chl^r^, Tet^r^, blank vector for gene complementary	Rose, 1988
pACYC184*phns*	*hns*::Chl^r^, pACYC184 with inserted IncX3 plasmid-encoded *phns* gene	This work

Strains J330(*phns*), J330(pACYC184), Pkhns(*phns*), and Pkhns(pACYC184) were obtained by transforming the corresponding plasmids into these strains by electrotransformation. Bacteria were stored in 25% glycerol medium at −80°C and recovered and cultured in Luria broth (LB) medium (containing 10 g NaCl, 10 g tryptone, and 5 g yeast per liter) at 37°C unless otherwise mentioned.

### Conjugation Experiments

Conjugation assays with pGZIncX3 and its derivatives were conducted as previously reported (Forns et al., 2005; Wang et al., 2017), using rifampin-resistant *E. coli* Ec600 as a recipient strain. Briefly, cultures of donor and recipient strains were grown overnight with shaking at different temperatures (25, 30, 37, or 42°C) in LB medium. The mating mixture, with a 1:2 donor to recipient ratio, was incubated at the corresponding temperature, without shaking overnight and then plated in LB agar supplemented with rifampin (200 μg ml^−1^) single (for recipients) or with meropenem (2 μg ml^−1^; for transconjugants). The plates were incubated at 37°C, and the mating frequency was calculated as the number of transconjugants per recipient.

### Plasmid Stability Test

Plasmid stability was determined as previously reported with minor modifications (Gao et al., 2020). Briefly, cultures were incubated at 25, 30, 37, or 42°C in a shaking water bath (200 rpm) and were diluted 1,000-fold in antibiotic-free LB broth. After 24 h, cultures were serially diluted and plated on antibiotic-free LB agar and LB agar containing meropenem (0.5 μg ml^−1^ L). The retention rate of the *bla*_NDM-1_ gene was calculated by dividing the number of colonies that grew on meropenem-containing LB agar by the number of colonies on antibiotic-free LB agar. In addition, 10 colonies were randomly selected and subjected to PCR validation of the *bla*_NDM-1_ gene. [It is unlikely that the chromosomal integration of *NDM* gene in plasmid stability test occurred. In our previous published work for Inc*X3* plasmid (Liu et al., 2020), we have proved that “PCR tests revealed that the blaNDM-1 gene was maintained by transconjugants and wild-type isolates for 1,000 generations. In addition, the location of the blaNDM-1 gene did not change in the decedents, as shown by Southern blotting.” The 24 h passage in this plasmid stability test could not lead to *NDM*-1 chromosomal integration].

### Competition Experiments

To assess the cost of carriage of *bla*_NDM_-bearing plasmid pGZIncX3 in *E. coli* at various temperatures (25, 30, 37, or 42°C), growth competition experiments between *E. coli* J53 and pGZIncX3-carrying derivatives were performed in LB broth using a previously published protocol (Johnson et al., 2015). Briefly, colonies from each strain were initially grown individually in LB broth at 25, 30, 37, or 42°C overnight with shaking. On day 0, bacteria were pelleted and suspended in equal volumes of PBS, and then inoculated at a volume of 25 μl each for competing strains into 5 ml of sterile LB broth. Competitions were performed for 24 h at each temperature, after which the cultures were serially diluted and plated on LB agar without antibiotic for total bacterial counts and on LB agar containing meropenem (0.5 μg ml^−1^) to select for cells containing pGZIncX3. All competition experiments were performed a minimum of three times. Fitness cost was calculated as follows: log2(*a*/*b*)/log2(*c*/*d*), where *a* = the number of plasmid-free cells at 24 h, *b* = the number of plasmid-free cells at 0 h, *c* = the number of plasmid-containing cells at 24 h, and *d* = the number of plasmid-containing cells at 0 h (Johnson et al., 2015).

### Plasmid Construction

For performing complementation experiments, the plasmid-encoded *phns* gene (positions 28,899–29,354 in GenBank accession JX104760) of IncX3 plasmid pNDM-HN380 (JX104760) was cloned into the pACYC184 vector. After amplifying the *phns* gene using PrimeSTAR^®^ HS DNA Polymerase (Takara), the PCR fragment was purified using TaKaRa MiniBEST DNA Fragment Purification Kit (Takara) and digested with EcoRI and BamHI restriction enzymes (Takara). Ligation was performed in pACYC184 digested with the same restriction enzymes. The resulting plasmid (pACYC184*phns*) was transformed into *E. coli* DH5α and picked in the presence of chloramphenicol. Primers were used for sequencing to confirm correct in-frame insertion of the *phns* gene.

### Real-Time Polymerase Chain Reaction

RNA of all samples was isolated using the E.Z.N.A. Bacterial RNA Kit (Omega Bio-tek). The qPCR was conducted with primers targeting the chromosome-encoded *idnT* (reference gene) and the plasmid gene as below. The isolated RNA was then transcribed into cDNA using a PrimeScript^™^ RT Master Mix (TaKaRa). Real-time polymerase chain reaction (RT-PCR) was used to quantify the expression of genes related to conjugation (*pilX4*, *pliX5*, *pilX9*, and *pilX10*). RT-PCR was conducted using the TB Green^™^ Premix Ex Taq^™^ II (TaKaRa) with the standard procedure for two-step PCR amplification (LightCycler96, Roche, Switzerland).

### Data Analysis

GraphPad Prism 8.0 was used for statistics analysis and figure drawing. ANOVA was used to analyze the difference between groups.

## Results

### The Transfer of the IncX3 Plasmid pGZIncX3 to *E. coli* Is Regulated by Temperature

**Figure 1:**
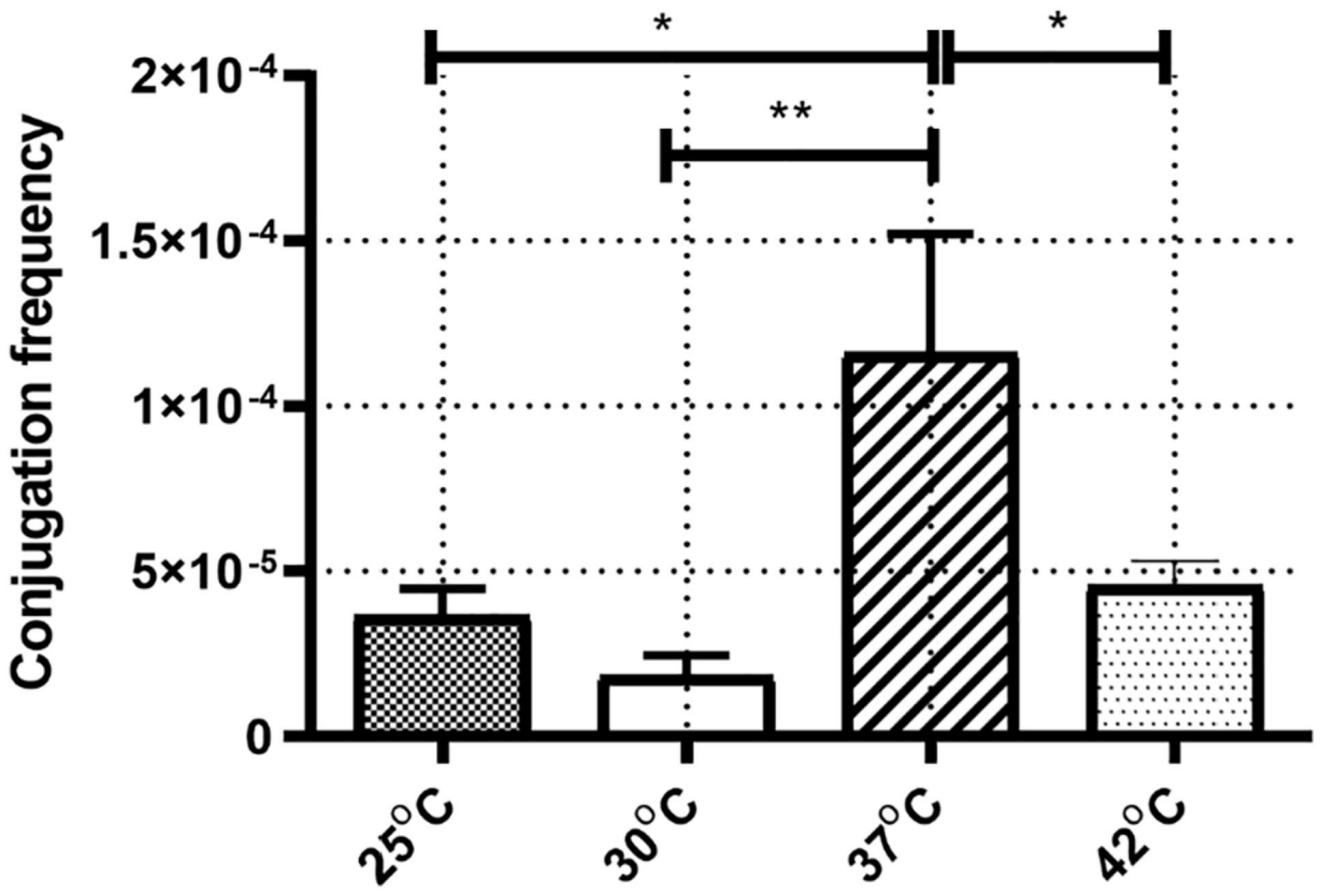
The conjugation frequency of IncX3 at four corresponding temperature. The conjugation frequency was calculated as transconjugants per recipient. Error bars indicate the standard deviations for six triplicate samples. ^*^p < 0.05/^**^p < 0.01 versus the result for conjugation frequency at 37°C.

The frequencies of IncX3 plasmid pGZIncX3 conjugative transfer from J330 to *E. coli* Ec600 were determined at different temperatures (Figure 1). ANOVA analysis showed a significant difference between conjugation frequencies at different temperatures (*p* = 0.002). Conjugation frequency was highest at 37°C (1.2 × 10^−4^) while incubation at 25°C, 30°C, and 42°C reduced transfer rates (3.7 × 10^−5^, 1.8 × 10^−5^, and 4.6 × 10^−5^, respectively) with the lowest conjugation frequencies at 25°C or 30°C. There was no significant difference between conjugation frequency at 25°C and 30°C.

### H-NS Gene Expression Varied With Differences in Temperature

**Figure 2:**
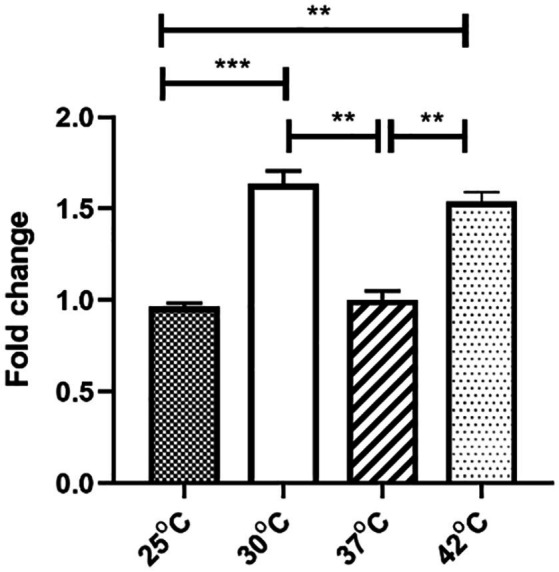
The effect of four temperatures on pGZIncX3 plasmid gene *phns* expression levels. Variances between different temperature groups were shown with ^*^(*p* < 0.05), ^**^(*p* < 0.01), and ^***^(*p* < 0.001).

To study the mechanism of the temperature dependence of plasmid conjugation, stability, and fitness, we focused on the pGZIncX3 gene *phns*, which encodes a H-NS-like protein. We previously showed that this gene is an inhibitor of plasmid transfer and partitioning (Liu et al., 2020). To further characterize the role of *phns,* we first determined the expression of *phns* under different temperatures, which revealed that *phns* was more highly expressed at 30°C and 42°C that at 25°C and 37°C (Figure 2). To avoid the impact of plasmid copy numbers (PCN) at different degree on gene expression, we compared them and found no difference (see Supplementary data 1, Figure n1). Moreover, *phns* itself did not affect PCN (Supplementary data 1, Figure n2).

### Plasmid-Encoded H-NS Affects Thermoregulation of IncX3 Transfer

**Figure 3:**
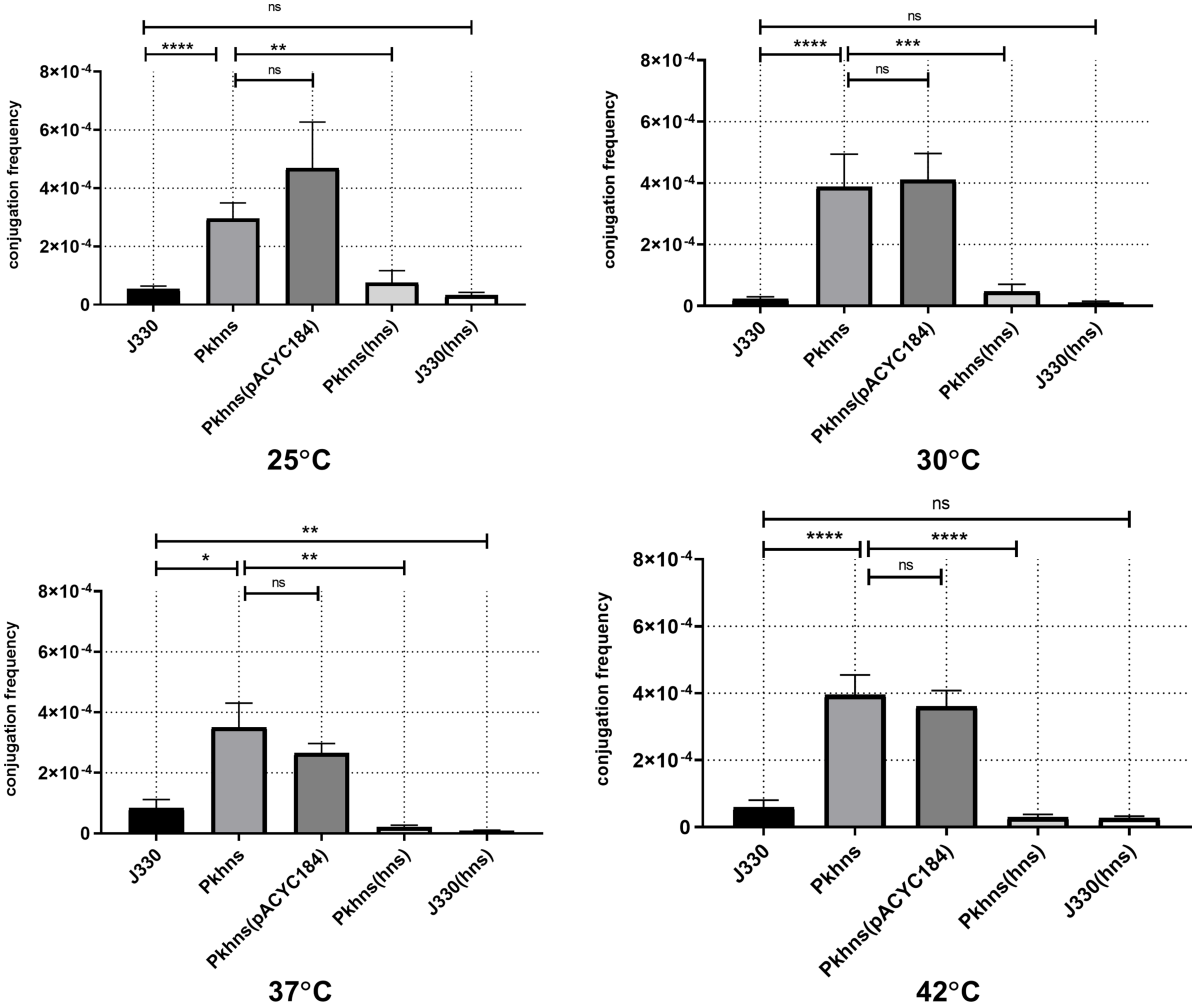
Plasmid-encoded H-NS-like protein represses plasmid transfer at different temperatures. Conjugation frequency at various temperature was evaluated for J330, Pkhns(*phns*-null strains), corresponding *phns* complementary strains Pkhns(hns), J330(hns), and corresponding blank-vector complementary strains J330(pACYC184), Pkhns(pACYC184). Variances between different temperature groups were shown with ^*^(*p* < 0.05), ^**^(*p* < 0.01), and ^***^(*p* < 0.001), ^****^(*p* < 0.0001).

To confirm the role of *phns* gene in plasmid fitness, we obtained pGZIncX3 derivatives harboring a *phns* deletion and the complemented mutant. *E. coli* strain J53 harboring either wild-type (WT) pGZIncX3 or its *phns* deleted or complemented derivatives were used as the donor in mating assays performed at four different temperatures (Figure 3).

At 37°C, conjugation results showed that *phns* deletion derepressed plasmid transfer. Lack of *phns* gene promoted the transfer rates of pGZIncX3 with the *phns* deletion (Pkhns) to Ec600 by 4.2-fold. Complementation of *phns* gene restored the transfer rates of Pkhns. We also found a reduction of conjugation frequencies in J330 complemented with *phns*.

At 25, 30, and 42°C, the plasmid-encoded H-NS-like protein also exerted a negative impact on conjugation. At every temperature, J330 with *phns* complementation exhibited decreased transfer rates relative to the parental strain J330 while *phns*-null strains showed robust transfer ability with high conjugation rates.

### Temperature Regulates the Conjugative Transfer of IncX3 Plasmid by Regulating the Expression of H-NS-Like Protein

**Figure 4:**
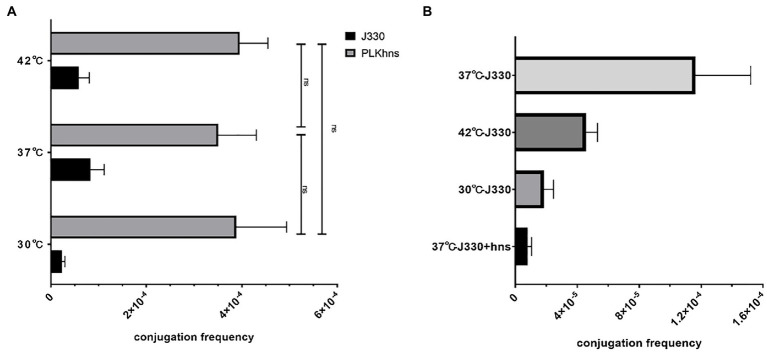
**(A)** Comparison of the frequency of conjugative transfer between the *phns* gene deletion plasmid and the original plasmid at different temperatures. At different temperatures, the joint frequency of pGZIncX3Δhns and pGZIncX3. **(B)** Comparison of conjugation frequency of pGZIncX3 at three different temperatures and 37 degrees when *phns* gene was overexpressed.

In order to verify that temperature affects the conjugation frequency of plasmids by regulating the expression of the *phns* gene, we compared the conjugation frequency of pGZIncX3 and pGZIncX3Δhns to the recipient strain EC600 at different temperatures by using a plasmid conjugation experiment. As shown in Figure 4A, when the *phns* gene is deleted, there is no difference in the conjugation frequency of plasmid pGZIncX3Δhns at 30 degrees, 37 degrees, and 42 degrees. Figure 4B shows the comparison of the conjugation frequency of pGZIncX3 in J330 at 30 degrees, 37 degrees, and 42 degrees with the conjugation frequency of pGZIncX3 in the *phns* gene overexpression strain J330 (*phns*) at 37 degrees. The frequency is significantly reduced at 30 degrees and 42 degrees, which is consistent with the decrease in the conjugation frequency of pGZIncX3 when the *phns* gene is overexpressed at 37 degrees, suggesting that *phns* gene is upregulated at 30 degrees and 42 degrees, further inhibiting plasmid conjugative transfer.

### Transcription of Several Genes Encoded in pGZIncX3 Is Enhanced in a *phns* Deletion Mutant

**Figure 5:**
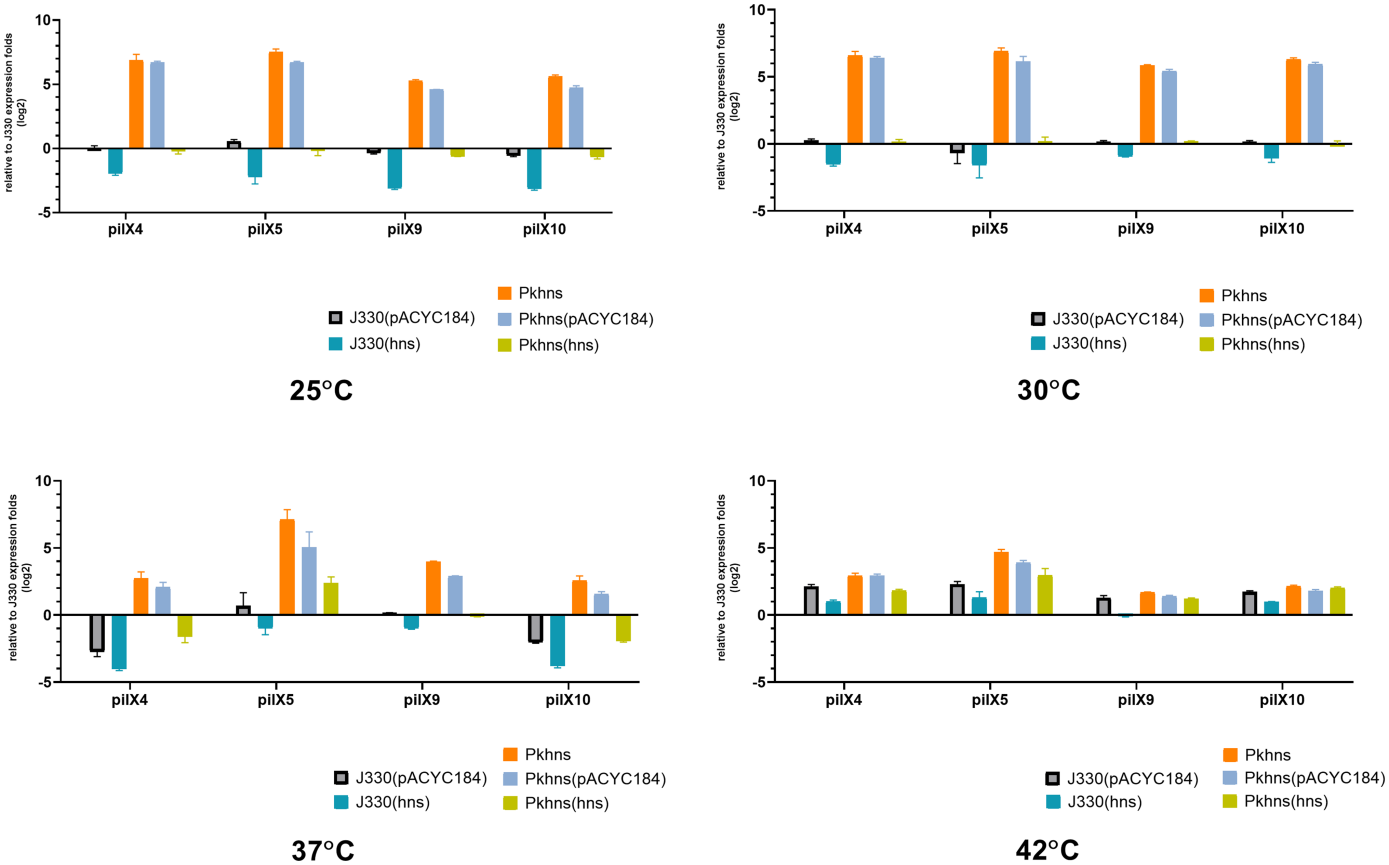
At four different temperatures (25, 30, 37, and 42°C), the mRNA expression of T4SS-related gene pilX4, 5, 9, and 10 of six gene context-related strains: *J330, Pkhns*(*phns*-null strains), corresponding *phns* complementary strains *Pkhns(hns), J330(hns),* and corresponding blank-vector complementary strains *J330(pACYC184), Pkhns(pACYC184)*.

We used RT-PCR to test if the plasmid-encoded H-NS-like protein has an impact on the transcription of plasmid genes involved in conjugation. In our previous work (Liu et al., 2020), we have analyzed the transcriptome differences between J330 and Pkhns, which showed that the expressions of all genes from *pilX* operon (including *pilX1,2,3,4,5,6,8,9,10,11*) are elevated in Pkhns. These genes encode constituents of a type IV secretion system (T4SS) and have been shown to be involved in conjugation (Chen et al., 2009). The *pilX4*, *pilX5*, *pilX9,* and *pilX10* genes were chosen to represent expression of the *pilX* operon at different temperatures (Figure 5).

We found that the *phns* gene reduces the expression of conjugation genes at various temperature which is corresponding to the results of conjugation test above. At low temperature (25°C or 30°C), the expression of *pilX4,5,9,10* in Pkhns could be more than 50-fold upregulated compared with J330. This experiment demonstrated that although low temperature could inhibit conjugation and the expression of genes involved in conjugation, the deletion of the plasmid gene encoding the H-NS homolog could abolish the impact of temperature.

### The Impact of Temperature on Plasmid-Mediated Bacterial Fitness and Plasmid Stability in *E. coli J53*

**Figure 6:**
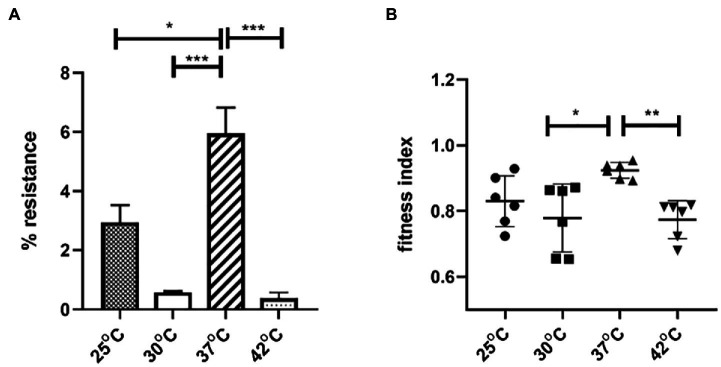
The impact of temperatures on IncX3 plasmid stability **(A)** and fitness cost **(B)** in *Escherichia coli*. **(A)** Plasmid stability is estimated by the percent of plasmid-containing *E. coli* at 24 h as determined by resistance to meropenem. **(B)** Fitness index is calculated by formula described in Section “Materials and Methods.” The higher fitness index represents better fitness of plasmid-containing strains. Significant differences between temperatures are indicated with asterisks: ^*^p < 0.05, ^**^p < 0.01, and ^***^p < 0.001.

To explore the impact of temperature on plasmid stability and *E. coli* host fitness, pGZIncX3 was transferred into *E. coli* J53 by conjugation to create J330. The stability of pGZIncX3 in J330 was evaluated after growth in antibiotic-free medium (Figure 6). pGZIncX3 was most stable at 37°C, with plasmid retention rate of 6.0%, with significantly lower plasmid retention at 25°C, 30°C, and 42°C (Figure 6A).

To evaluate the impact of pGZIncX3 on J330 fitness at different temperatures, growth competition experiments between J330 and plasmid-free J53 were performed at different temperatures. pGZIncX3 had a higher fitness cost at 30°C and 42°C than at 37°C (Figure 6B), but no significant difference in fitness cost was observed between 25°C and 37°C.

### The Impact of *phns* on Plasmid Stability and Host Fitness Cost at Various Temperature

**Figure 7:**
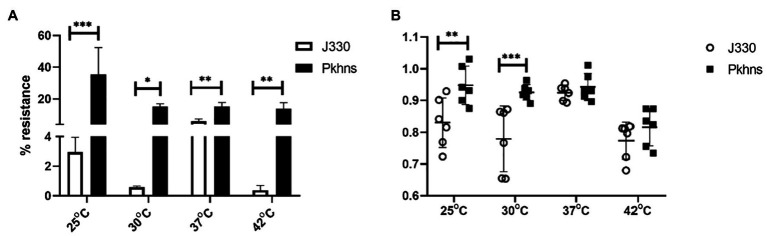
At various temperature, the impact of plasmid-encoded H-NS-like protein on IncX3 plasmid stability **(A)** and fitness cost **(B)** in *E. coli*. **(A)** Plasmid stability is estimated by the percent of plasmid-containing *E. coli* at 24 h (% resistance). **(B)** Fitness index is calculated by formula described in Section “Materials and Methods.” The higher fitness index represents better fitness of plasmid-containing strains. Significant differences are indicated with ^*^(*p* < 0.05), ^**^(*p* < 0.01), and ^***^(*p* < 0.001).

To test if plasmid stability also changed through regulation of the plasmid-encoded H-NS protein, we compared stability of pGZIncX3 in the *E. coli* host at a range of temperature. Under all temperatures tested, *phns*-null J330 derivate strains showed higher plasmid retention rates versus wild-type strains (Figure 7A).

We also tested the effects of *phns* on bacterial fitness. Deletion of *phns* slightly increased the fitness of plasmid-containing *E. coli* fitness at 25 and 30°C, whereas overall fitness at 37 and 42°C did not change significantly (Figure 7B).

## Discussion

The role of *bla*_NDM_-bearing IncX3 plasmids in the spread of carbapenem resistance makes them an important threat to global public health. Our results show that the phenotypes conferred by the IncX3 plasmid pGZIncX3 are affected by environmental temperature and the plasmid-encoded H-NS-like protein is involved in this regulation. In this study, we focused on the effects of four distinct temperatures on IncX3 plasmid phenotypes as they are representative of environmental temperatures in many countries in Asia and the Middle East (25°C and 30°C), the temperature of the human body (37°C), and the body temperature of chickens (42°C; Rozwandowicz et al., 2019).

Here, we demonstrate that the IncX3 plasmid pGZIncX3 could transfer at highest frequencies at 37°C, suggesting that transfer of this important group of plasmids is highest in the guts of humans and other mammals, which is in line with previous work (Liakopoulos et al., 2018; Wang et al., 2018), that showed that the optimal temperature for conjugative transfer of IncX3 plasmids was 37°C. In addition, we have shown that at 37°C, pGZIncX3 was most stable in the absence of antibiotic selective pressure and imposed the lowest fitness costs. It is not surprising that conjugation rates and stability properties are both optimized at 37°C. As there is an evolutionary selection for small fitness effects of plasmids to ensure long-term persistence of the plasmids in the absence of selective pressures (Wein et al., 2019), these plasmids have adapted to life in gut commensals of mammals.

We found a new mechanism for IncX3 plasmid adaptation to different temperatures. We explored the potential mechanisms that link environmental temperature to plasmid stability, plasmid transfer, and fitness costs of pGZIncX3. Our study showed that growth at 30°C and 42°C caused the elevated expression level of the pGZIncX3 *phns* gene and we confirmed the role of this gene in suppressing conjugation in a temperature-dependent fashion. This phenomenon was linked to *phns-*dependent repression of the genes found in the pilX operon that encodes the IncX3 T4SS, namely, *pilX4*, *pilX5*, *pilX9*, and *pilX10*.

We also found that plasmid stability is repressed by *phns* under all temperatures tested. H-NS inhibition of conjugation leads to lower stability through the mechanisms mentioned above. Results in our study revealed the IncX3 plasmid-encoded H-NS-like protein only slightly affects host fitness, in line with previous work on a different IncX3 plasmid-encoded *hns*-like gene (Ho et al., 2013). But, in previous work, the IncHI1 plasmid-encoded Sfh protein, which is an H-NS homologue, was proposed to promote plasmid transfer to new bacterial hosts with little effects on bacterial fitness (Doyle et al., 2007). The discordant observations between these two plasmid-encoded H-NS homologs could be due to differences in host species (*Salmonella* spp. vs. *E. coli*), plasmid length (54 vs. 100 kbp), and host adaptive mutations (Takeda et al., 2011). Moreover, discordance of plasmid-encoded *hns* function between *E. coli* and *Salmonella* may be explained by significantly different *hns*-related transcription profiles as shown in the Doyle *et al* study (Doyle et al., 2007) and our previous work (Liu et al., 2020). The current consensus is that an increase in temperature (from 30°C to 37°C) disrupts the binding of H-NS to DNA, resulting in the derepression of gene expression (Shahul Hameed et al., 2019) and this would explain the phenotypes observed in our study. Interestingly, the gene encoding the widely studied H-NS-like protein StpA exhibited higher expression levels at 42°C compared to 37°C which is in line with our findings (Muller et al., 2010). Surprisingly, we saw little difference in plasmid-mediated phenotypes between 25 and 37°C, which suggest that regulatory pathways other than *phns* are important for the regulation of plasmid traits at 25°C. Thus, our results proved that the variation of H-NS expression contributes to the different phenotypes of plasmids at 30°C, 42°C, and 37°C.

The optimal function of IncX3 plasmids at 37°C contrasts with that of IncHI1 plasmids like R27, which exhibit highest transfer frequencies at approximately 25°C. In both cases, thermoregulation is exerted by plasmid-encoded H-NS-like proteins. This suggests that different plasmid families have adapted to certain environmental conditions experienced by their hosts, and it will be interesting to determine whether transfer frequencies are temperature sensitive. It seems possible that the thermoregulation of plasmid functions influences the contributions of certain plasmid types to the dissemination of antibiotic resistance determinants in different environments. For example, IncHI1 plasmids might be expected to play a greater role in the transfer of resistance genes within environmental bacterial populations experiencing ambient temperatures of approximately 25°C, while IncX3 plasmids play a major role in the spread of resistance genes within mammalian enteric populations.

We acknowledge that our study has several limitations. First, our investigation focused on the plasmid-encoded gene *phns* to examine its modulation of plasmid characteristics under different temperatures. However, we could not take the interactions between host and plasmid regulators into consideration. For example, it has been reported that in *E. coli* chromosome-encoded temperature-sensor σ-32 is also involved in thermoregulation of plasmid conjugation (Rozwandowicz et al., 2019). Secondly, although we found that at 30 and 42°C, IncX3 plasmid-bearing *E. coli* exhibits lower transfer ability and stability, various IncX3 plasmids have been isolated from natural environments (Cheng et al., 2019). Therefore, we speculate that IncX3 plasmid-bearing host might require compensatory evolution for long-term adaptation to new environments with temperatures that are different to the mammalian gut (Zwanzig et al., 2019). Further studies of plasmid-host co-evolution need to be conducted to explore the mechanism by which plasmids and hosts reach a fitness optimum at various temperature.

## Conclusion

In conclusion, this report describes the differential impact of physiological and environmental temperature on IncX3 plasmid phenotypes. The IncX3 plasmid studied here exhibited a higher transfer frequency, was more stable and imposed a lower fitness cost at 37°C than at other temperatures. Temperature-regulated variation involve a plasmid-encoded H-NS-like protein, which acts as a thermo-sensor and is upregulated at 30 and 42°C. The H-NS-like protein inhibits conjugation genes and appears to decrease plasmid stability and host fitness. Our findings suggest that *bla*_NDM_-bearing IncX3 plasmids have evolved to persist and transfer optimally in the mammalian gut, which will complicate the control of the spread of carbapenem-resistant pathogens.

## Data Availability Statement

The original contributions presented in the study are included in the article/Supplementary Material, and further inquiries can be directed to the corresponding authors.

## Author Contributions

All authors listed have made a substantial, direct, and intellectual contribution to the work, and approved it for publication.

## Funding

The research was supported by the National Natural Science Foundation of China (81772238), the Project of International Cooperation and Exchanges NSFC (8181101332), and the Guangzhou Municipal Science and Technology Bureau (project no. 201607020044) and the Medical Research Council (MR/S013660/1).

## Conflict of Interest

The authors declare that the research was conducted in the absence of any commercial or financial relationships that could be construed as a potential conflict of interest.

## Publisher’s Note

All claims expressed in this article are solely those of the authors and do not necessarily represent those of their affiliated organizations, or those of the publisher, the editors and the reviewers. Any product that may be evaluated in this article, or claim that may be made by its manufacturer, is not guaranteed or endorsed by the publisher.
