# Supplementary material for: Temperature-Regulated IncX3 Plasmid Characteristics and the Role of Plasmid-Encoded H-NS in Thermoregulation
Source: Front Microbiol. 2022 Jan 6;12:765492. doi: 10.3389/fmicb.2021.765492 (PMC8770905; doi:10.3389/fmicb.2021.765492)
Supplement: Supplementary file 1 [file Data_Sheet_1.pdf]

The copy numbers of plasmid on gene expression.

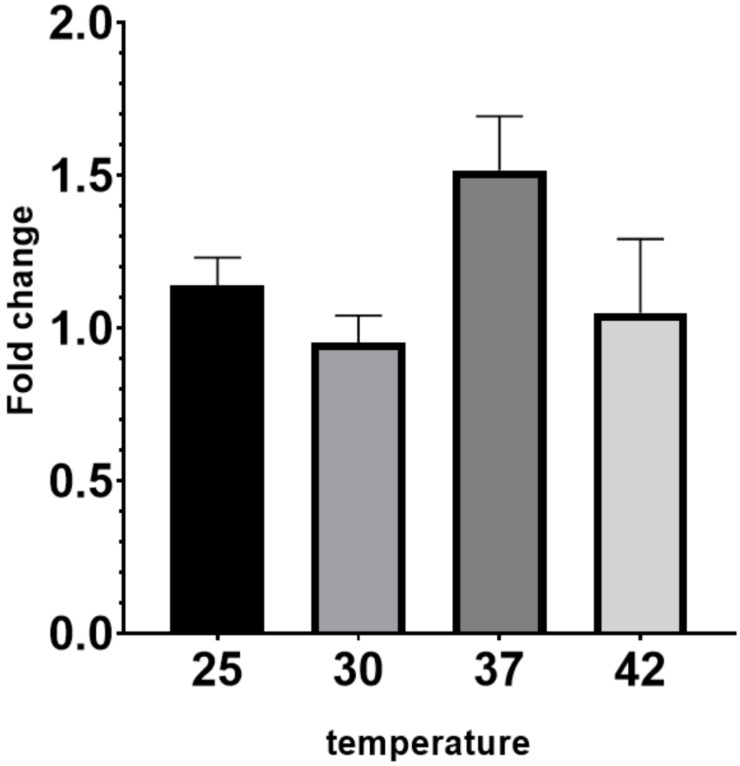

Figure n1 Plasmid copy number（PCN） at 25,30 and 42 showed similar level (around 1). PCN at 37 was slightly higher than that at other temperature but did not reach 2 copy. Given that the hns gene expression level at 30 and 42 degrees is higher than 37 degrees, we do not think it is the result of an increase in PCN.

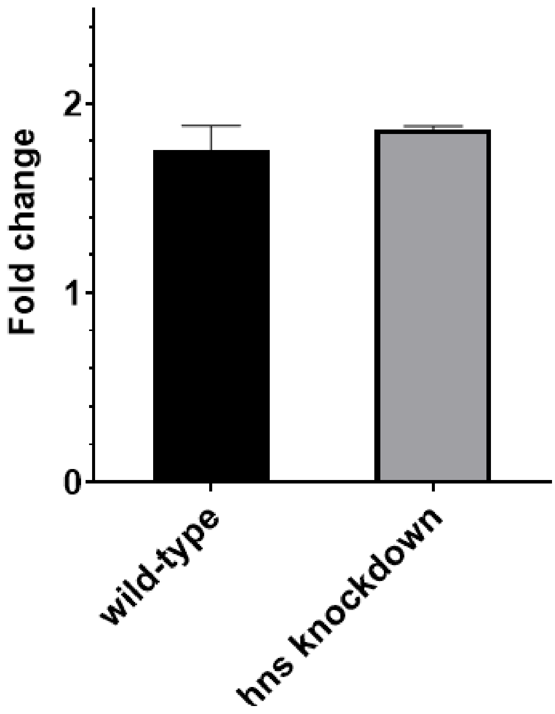

Figure n2 PCN of hns knockdown IncX3 showed no difference with wild type IncX3 plasmid. H-NS does not affect the plasmid replication and PCN. (The results of the transcriptome regulated by the hns gene also confirm this view, which was reported in our previous work. Ref 12)
